# Optimal pricing and marketing planning for deteriorating items

**DOI:** 10.1371/journal.pone.0172758

**Published:** 2017-03-17

**Authors:** Seyed Reza Moosavi Tabatabaei, Seyed Jafar Sadjadi, Ahmad Makui

**Affiliations:** Department of Industrial Engineering, Center of excellence in optimization and manufacturing, Iran University of Science and Technology, Tehran, Iran; Chongqing University, CHINA

## Abstract

Optimal pricing and marketing planning plays an essential role in production decisions on deteriorating items. This paper presents a mathematical model for a three-level supply chain, which includes one producer, one distributor and one retailer. The proposed study considers the production of a deteriorating item where demand is influenced by price, marketing expenditure, quality of product and after-sales service expenditures. The proposed model is formulated as a geometric programming with 5 degrees of difficulty and the problem is solved using the recent advances in optimization techniques. The study is supported by several numerical examples and sensitivity analysis is performed to analyze the effects of the changes in different parameters on the optimal solution. The preliminary results indicate that with the change in parameters influencing on demand, inventory holding, inventory deteriorating and set-up costs change and also significantly affect total revenue.

## Introduction

During the past two decades, there have been considerable efforts devoted to achieving profitability through pricing strategies. Indeed, companies can increase their market shares and maximize customer satisfaction through systematic pricing strategies. The primary concern with many optimal pricing strategies is to determine appropriate pricing to maximize profitability. Pricing strategy also determines how customers view and respond to different products or services. Appropriate pricing strategy depends on how to position products or services. Optimal pricing also helps competitors for developing pricing strategies. In fact, the key point in a competitive environment is associated with marketing and production decisions. Generally, three main strategies have been introduced for production and marketing sectors: Separate strategy, Joint strategy and coordinated strategy. The recent progress on pricing and marketing strategies leads to motivation increase for having more realistic optimal lot-sizing strategies.

In recent years, insufficient resources, increase on expenses and shorter life cycles of products have created motivation to plan for more integrated supply chain. Under such circumstances, pricing has become one of the most important factors on supply chain planning.

Lee and Kim [1] considered full and partial models of a joint production and marketing problems in order to maximize the profitability facing constant, but price and marketing dependent demand over a planning horizon to determine price, marketing costs and lot-size, simultaneously. Geometric programming method was utilized to solve and obtain managerial results for a pricing model in their approach. Kim and Lee [2] investigated the fixed and variable capacity problems of jointly determining an item’s price and lot size for a profit-maximizing firm with a sophisticated non-linear demand function which was a function of selling price. Chen [3] introduced a profit-maximizing inventory model for joint optimization of quality level, selling quantity and purchasing price of a product for intermediate firms by assuming that the selling price, supply rate of the product and the fixed selling costs were power functions of decision variables. Also the resultant model was solved through geometric programming (GP) method in the mentioned research work. Kochenberger [4] tackled the non-linear EOQ problem through GP (Duffin et al. [5]) method. Cheng [6] implemented GP for solving a lot-sizing problem where the cost per unit was a function of demand. Lee [7] developed GP formulations for an EOQ problem where lot-size and selling prices were decision variables and the cost was considered to be a function of the lot-size. Abuo-El-Ata et al. [8] introduced a multi-item production lot-size inventory model with a varying order cost and solved the resultant model by GP technique. Mandal et al. [9] solved multi-objective fuzzy inventory model with three constraints by GP approach. Jung and Klein [10] considered the difference between the optimal order quantities via GP technique by comparing the cost minimization model to the profit-maximization model. Sadjadi et al. [11] suggested a profit maximizing GP model for optimal production and marketing planning where demand depends on price and marketing expenditures and production cost is inversely associated with the lot-size. Also a flexible production rate and derive a closed-form solution for the problem were developed in their approach. Parlar and Weng [12] described a coordinating pricing and production decisions based on price competition. They presented a method based on GP to find the optimal solution. Liu [13] proposed a discount model in a profit maximization problem and suggested a method based on GP to find the optimal solution. Fathian et al. [14] explained a pricing model for electronic products and presented a mathematical model by assuming that demand was a function of product’s price, marketing and service expenditures, and unit production cost was a function of demand. To determine the optimal solution for the problem, they used the GP dual method. Sadjadi et al. [15] addressed a production-marketing problem in the context of the unreliable production process. They used the non-linear posynomial GP and obtained a closed form solution for jointly optimized the lot-size, marketing expenditures, set-up cost, and reliability of the product. Ghosh and Roy [16] reformulated a goal programming problem by using GP technique. In this work, retailers provide a quality service which could pose higher prices as the competition increments (Venkatesan et al. [17]). Chun and Kim [18] examined pricing strategies between ordinary logged off firms and online firms to discover which item is higher in cost contingent indifferent channels. Dai et al. [19] described the pricing strategies of multiple firms giving the same service in the rivalry for a common pool of clients where demand on every firm is a linear function of price. The collaborations among products, retailers and market characteristics were analyzed by Venkatesan et al. [17]. They developed a multi-level hierarchical linear model and described that service quality positively influences on retail value levels. Gupta et al. [20] concentrated on a few concepts for retail pricing strategies and described that the risk neutral consumers could not be generally more prone to lean on the electronic channel than risk–averse customers could. Aron et al. [21] demonstrated that as purchasers modify their product choices, accordingly of better demand agent technologies, seller’s revenues decrease since the gains from better buyer information is ruled by the bringing down of the total value created from the transactions. Polatoglu [22] considered optimal pricing and procurement decisions with a one-period pure inventory model under deterministic or probabilistic demand. Sajadieh et al. [23] concentrated on a two phase supply chain comprising of one seller and one purchaser. They built up a coordinated production inventory marketing model for making appropriate decisions on the applicable profit-maximizing decision variable values. The proposed model depends on the joint total benefit of both the seller and the purchaser and determined the optimal ordering, shipment and pricing policies. Huang et al. [24] considered coordination of big business choices, including suppliers and component selection, pricing and inventory in a multi-level supply chain composed of multiple suppliers. The problem was modeled as a three-level dynamic non-cooperative game.

There are literally several items, which are under deterioration and it is important to set-up an appropriate model for inventory management of such products.

A heuristic model was presented by Bahari-Kashani [25] for deciding the ordering schedule when an inventoried item is liable to deteriorate and demand changes linearly over time. Chung and Lin [26] considered discounted cash flow (DCF) to deal with the inventory replenishment issue for deteriorating items taking into account the time value of money over a settled planning horizon. They created models and optimal solutions with complete backlogging and without backlogging and demonstrated that the total variable cost function was convex. An inventory model for deteriorating items with a deficiency occurring at the supplier level, including a supply chain between the producer and buyer was produced by Rau et al. [27]. The results of their study showed that integrated decisions were more cost-effective compared with independent decisions on the supplier, producer or buyer. Hou [28] introduced an inventory model for perishable items with a time-varying stock dependent demand under inflation. It is expected that the supplier offers a credit period to the retailer and the length of credit period was a function of order quantity. Moreover, the motivation behind the study was to minimize the present value of retailer's total cost. System dynamics thinking to propose a new order system and conduct a systematical simulation were applied by Lee and Chung [29]. The consequences of acceptance and model testing for constructing model demonstrated that system dynamics simulation methodology was a good solution strategy. Chung [30] explored the impacts of stock and warranty dependent demand and also considered the relationship between imperfect, warranty policy and inspection scheduling. Additionally two-stage production and inventory deteriorating model were integrated in the study for renewal strategy and assessment plan for producing utilized time-weighted inventory approach. The results demonstrated that settled offering rate, the holding cost and the unit inspection cost were the basic variables in applying the deteriorating inventory model. Pricing strategy plays an essential role on deteriorating items and it is important to look for appropriate optimal pricing for deteriorating items. There are several studies on this subject. Deterministic inventory models on quantity discount, pricing and partial back ordering, for instance were considered by Wee [31] when the product in stock deteriorates over time. An optimal pricing and ordering policy of a deteriorating item with price sensitive demand was developed by Yang [32]. Since it is more advantageous for the seller in compare with the purchaser when both the purchaser and the seller were incorporated, a quantity discount pricing strategy was important to lure the purchaser to acknowledge the organization together. Dye et al. [33] analyzed an infinite horizon single product economic order quantity where demand and deterioration rate area were continuous and differentiable function of price and time, individually. Moreover, they also considered shortages and completely backlogged. The problem of dynamic pricing, promotion and replenishment for a deteriorating item subject to the supplier’s trade credit and retailer’s promotional effort were studied by Tsao and Sheen [34]. They embraced a price and time dependent demand functions to model the finite time horizon inventory for deteriorating items and also tried to decide the optimal retail price, the promotional effort and the replenishment quantity so that the net profits would be maximized. Huang [35] built up an integrated inventory model to decide the optimal policy on states of order processing cost reduction and permissible delay in payments. Both, the seller and the purchaser used information technologies to reduce the common costs. The order processing cost can be lessened by certain expenditures and influence lot-size decisions. At the same time, the presence of the credit period serves to reduce the cost of holding stock for the purchaser, since it decreases the measure of capital invested in stock for the term of the credit period. Dye [36] investigated deterministic economic order quantity model with generalized type demand, deterioration and unit purchase cost functions under two levels of trade credit policies. The goal was to locate the optimal values of selling prices, replenishment number and scheme which maximize the total profit over the finite planning horizon. Joint pricing and inventory control model for items which have non-instantaneous deterioration rate were considered by Maihami and Nakhai [37]. They embraced a demand function which was a function of the price and time. The shortage was permitted and partially backlogged. The real target was to decide the optimal selling price, the optimal replenishment schedule and the optimal order quantity, simultaneously such that the total profit is maximized.

One of the primary assumptions of most lot sizing problems is that demand is considered as constant. In some cases, demand is assumed as a function of price and marketing expenditures, but in all cases, the problem statement was intentionally designed in a form of geometric programming with a limited number of difficulties, e.g. 0 or 1. Such problems are often unrealistic and impractical. The fundamental assumption of this paper is that the demand was influenced by price elasticity, marketing, and after-sales service and additionally assumes that the costs are affected by the demand function.

In this study, a new optimal pricing model is suggested for deterioration items by considering total revenue and the costs of production, marketing, set-up, inventory holding, inventory deteriorating and after-sales service and also shows that the cost of production has different trends depending on the production size. After-sales service quality, proper price and advertising are the key parameters for the proposed model because they have greater impacts on consumers' purchase decisions. This paper presents a mathematical model which deals with a problem with the relatively high degree of difficulty. The resultant problem is formulated as a parametric GP with five degrees of difficulty and it is solved using recent advances in optimization techniques.

The paper is supported by various numerical examples and the results are analyzed under different scenarios. This study is believed to be the first to solve optimal pricing problems by under real-world circumstances where the proposed model is formulated as a GP problem with a relatively high degree of difficulty.

The rest of this paper is organized as follows; in section 2, the problem statement and underlying assumptions for developing the profit maximization GP are presented. In section 3, mathematical formulations of the GP model is proposed and then the procedure to detect the optimal solution is given in section 4 and section 5. The implementation of the proposed model is described using some numerical example in section 6. Finally, Section 7 presents the conclusions and directions for future research.

### Nomenclature

The following notations are adopted for developing the mathematical model:

**Table:** 

*D*	Demand
*C*	Unit production cost
*i*	Cost of having one dollar of the item tied up in inventory for a unit time interval
*a*	Set-up cost of the production
*α*	Price elasticity to demand
*δ*	Marketing expenditure elasticity to demand
*σ*	After-sales service expenditure elasticity to demand
*Θ*	Deterioration rate
*h*	Holding cost per unit per unit time
*I (t)*	Inventory Level’s
(TR)	Total Revenue
(PC)	Production Cost
(MC)	Marketing Cost
(SC)	Set-up Cost
(IHC)	Inventory Holding Cost
(IDC)	Inventory Deteriorating Cost
(ASC)	After-Sales Service Cost

### Decision variable

**Table:** 

*P*	Selling price per unit
*M*	Marketing expenditure per unit
*Q*	Production lot-size (units)
*S*	After-sales service expenditure per unit
*T*	Duration of each period
Π (*P*, *Q*, *M*, *S*, *T*)	The profit function
(*)	Here (*) represents optimality for *P*, *Q*, *M*, *S*, *T* and Π.

## Assumptions

Here demand is a nonlinear function of price (*P*), marketing expenditure (*M*) and after-sales services (*S*) as follows

$$D=kP^{-\alpha }M^{\delta }S^{\sigma },$$ (1)

Where *α* > 1, 0 < *δ* < 1, 0 < *σ* < 1 and *k* is a scaling constant, *α* is price elasticity to demand (*α* > 1) (Lilien et al. [38]). Parameter *C* is defined as a production cost per unit. In this study, *C* is a unit cost and can be discounted with *β*. Therefore, we have,

$$C=r_{1}Q^{3}+r_{2}Q^{2}+r_{3}Q,$$ (2)

Where *Q* is production lot-size (units), *r* is the scaling constant for unit production cost. We assume that *r*_*1*_ < 0 and *r*_*3*_ <0. Note that the cubic form of the cost function is more realistic terms compared with previously used non-increasing exponential form.

In this model, no shortages are permitted and replenishment lead time is instantaneous and time horizon is infinite. It is also considered a single-product inventory system that deterioration rate is constant; where 0<*θ*<1.

Let *I* (*t*) be the product inventory level at any time *t* (0 ≤ *t* ≤*T*). At first, the stock level will be *Q*. Duration of each period [0, *T*], demand and deterioration deplete the inventory level; furthermore, the change of inventory level regarding time can be portrayed by the following differential equation:

$$\frac{dI\left(t\right)}{dt}+\theta I\left(t\right)=-D.\;0\leq t\leq T$$ (3)

With boundary conditions *I* (0) = *Q* and *I* (*T*) = 0, the solution of the differential Eq (3) is as follow:

$$I\left(t\right)=\frac{D}{\theta }\left(e^{\theta \left(T-t\right)}-1\right)\;0\leq t\leq T$$ (4)

Note that when *I* (0) = *Q*, then the production lot-size for the duration of each period is as follows,

$$Q=\frac{D}{\theta }\left(e^{\theta T}-1\right)$$ (5)

## The proposed model

This paper presents a mathematical model for a three-level supply chain, including a producer, a distributor, and a retailer. The proposed study considers the production of a deteriorating item where demand is influenced by Product selling prices (P*), marketing expenditure (M*), Production lot-size (Q*) and after-sales service expenditure (S*) and duration of each period (T*). In the beginning of production schedule, the manufacturer produces a lot to meet the demand of the cycle and the product deteriorates over time. In our model, the production cost is a non-decreasing function of lot-size. Therefore, the problem is formulated as follows:

Total Revenue (TR):

$$=PD=kP^{1-\alpha }M^{\delta }S^{\sigma }$$ (6)

In this paper, we assume the component of expenditure function includes: production, marketing, set-up, inventory holding, inventory deteriorating and after-sales service expenditures. These components are defined as follows:

I. Annual Production Cost (PC):
  $$=CD=kr_{1}P^{-\alpha }M^{\delta }S^{\sigma }Q^{3}+kr_{2}P^{-\alpha }M^{\delta }S^{\sigma }Q^{2}+kr_{3}P^{-\alpha }M^{\delta }S^{\sigma }Q$$ (7)
II. Annual Marketing Cost (MC):
  $$=MD=kP^{-\alpha }M^{\delta +1}S^{\sigma }$$ (8)
III. Annual Set-up Cost (SC):
  $$=\frac{aD}{Q}=kaP^{-\alpha }M^{\delta }S^{\sigma }Q^{-1}$$ (9)
IV. Annual Inventory Holding Cost (IHC):
  $$=\frac{h}{T}\int_{0}^{T}I\left(t\right)dt=\frac{hD}{T\theta }\int_{0}^{T}\left(e^{\theta \left(T-t\right)}-1\right)dt=\frac{hD}{T\theta^{2}}\left(e^{\theta T}-\theta T-1\right)$$ (10)
  Taylor series can be expressed as follows for small amount of *θ*:
  $$e^{\theta T}≅1+\theta T+\frac{\theta^{2}T^{2}}{2}.$$ (11)
  Therefore, the inventory holding cost function is as follows:
  $$\text{IHC}=2^{-1}hkP^{-\alpha }M^{\delta }S^{\sigma }T.$$ (12)
  On the other hand, holding cost can be defined as a function of production cost as follows:
  H=ic (13)
  Finally, the inventory holding cost can be written as follows:
  $$\text{IHC}=2^{-1}ir_{1}Q^{3}kP^{-\alpha }M^{\delta }S^{\sigma }T+2^{-1}ir_{2}Q^{2}kP^{-\alpha }M^{\delta }S^{\sigma }T+2^{-1}ir_{3}QkP^{-\alpha }M^{\delta }S^{\sigma }T$$ (14)
V. Annual Inventory Deteriorating Cost (IDC):
  $$=\frac{P}{T}\int_{0}^{T}\theta I\left(t\right)dt=\frac{PD}{T}\int_{0}^{T}\left(e^{\theta \left(T-t\right)}-1\right)dt=\frac{PD}{T\theta }\left(e^{\theta T}-\theta T-1\right)$$ (15)
  Using Taylor series approximation, annual inventory deteriorating cost can be written as follows:
  $$\text{IDC}=2^{-1}kP^{1-\alpha }M^{\delta }S^{\sigma }\theta T$$ (16)
VI. Annual After-Sales Service Cost (ASC):
  $$=SD=kP^{-\alpha }M^{\delta }S^{\sigma +1}$$ (17)
  Profit function can be written based on revenue function, cost function, and mentioned assumptions as follows,
  $$\begin{array}{l} \text{Max}\;\Pi \left(P,Q,M,T,S\right)\\ \;=\text{Total Revenue}-\text{Production Cost}-\text{Marketing Cost}-\text{Set}\_\text{up Cost}\\ \;-\text{Inventory Holding Cost}-\text{Inventory Deteriorating Cost}\\ \;-\text{After}\_\text{Sales Service Cost}\\ \text{Max}\;\Pi \left(P,Q,M,T,S\right)=\text{TR}-\text{PC}-\text{MC}-\text{SC}-\text{IHC}-\text{IDC}-\text{ASC} \end{array}$$ (18)

This particular form of relationship is a signomial function, which can be converted into posynomial parametric GP problems.

The optimal solution of such a problem can be determined by GP method which is explained in details in the following section.

## Optimal solution of model

The mathematical function for model (18) is written as follows,

$$\begin{array}{l} \text{Max}\;\Pi \left(P,Q,M,T,S\right)\\ \;=kP^{1-\alpha }M^{\delta }S^{\sigma }+kr_{1}P^{-\alpha }M^{\delta }S^{\sigma }Q^{3}-kr_{2}P^{-\alpha }M^{\delta }S^{\sigma }Q^{2}+kr_{3}P^{-\alpha }M^{\delta }S^{\sigma }Q\\ \;-kP^{-\alpha }M^{\delta +1}S^{\sigma }-kaP^{-\alpha }M^{\delta }S^{\sigma }Q^{-1}+kr_{1}P^{-\alpha }M^{\delta }S^{\sigma }Q^{3}e^{\theta T}T^{-1}\theta^{-2}\\ \;-kr_{2}P^{-\alpha }M^{\delta }S^{\sigma }Q^{2}\theta^{-1}+kr_{3}P^{-\alpha }M^{\delta }S^{\sigma }QT^{-1}\theta^{-2}+kr_{1}P^{-\alpha }M^{\delta }S^{\sigma }Q^{3}\theta^{-1}\\ \;-kr_{2}P^{-\alpha }M^{\delta }S^{\sigma }Q^{2}\theta^{-1}+kr_{3}P^{-\alpha }M^{\delta }S^{\sigma }Q\theta^{-1}+kr_{1}P^{-\alpha }M^{\delta }S^{\sigma }Q^{3}T^{-1}\theta^{-2}\\ \;-kr_{2}P^{-\alpha }M^{\delta }S^{\sigma }Q^{2}T^{-1}\theta^{-2}+kr_{3}P^{-\alpha }M^{\delta }S^{\sigma }QT^{-1}\theta^{-2}\\ \;-kP^{1-\alpha }M^{\delta }S^{\sigma }e^{\theta T}T^{-1}\theta^{-1}+kP^{1-\alpha }M^{\delta }S^{\sigma }+kP^{1-\alpha }M^{\delta }S^{\sigma }T^{-1}\theta^{-1}\\ \;-kP^{-\alpha }M^{\delta }S^{\sigma +1} \end{array}$$ (19)

Let *n* and *m* be the number of terms and variables in the primal problem (19), respectively. Then the degrees of difficulty (*d*) for the resulting GP problem are *d* = *n* − (*m* +1).

We have *n* = 19 & *m* = 5. So there *d* = 19-(5+1) = 13.

We need to make some necessary modifications in order to change the problem in to a posynomial GP model and reduce the degrees of difficulty of the proposed model. For this purpose, we use a Taylor series approximation for annual inventory holding cost and annual inventory deteriorating cost.

So we have:

$$\begin{array}{l} \text{Max}\;\Pi \left(P,Q,M,T,S\right)\\ \;=kP^{1-\alpha }M^{\delta }S^{\sigma }+kr_{1}P^{-\alpha }M^{\delta }S^{\sigma }Q^{3}-kr_{2}P^{-\alpha }M^{\delta }S^{\sigma }Q^{2}+kr_{3}P^{-\alpha }M^{\delta }S^{\sigma }Q\\ \;-kP^{-\alpha }M^{\delta +1}S^{\sigma }-kaP^{-\alpha }M^{\delta }S^{\sigma }Q^{-1}+2^{-1}ir_{1}Q^{3}kP^{-\alpha }M^{\delta }S^{\sigma }T\\ \;-2^{-1}ir_{2}Q^{2}kP^{-\alpha }M^{\delta }S^{\sigma }T+2^{-1}ir_{3}QkP^{-\alpha }M^{\delta }S^{\sigma }T-2^{-1}kP^{1-\alpha }M^{\delta }S^{\sigma }\theta T\\ \;-kP^{-\alpha }M^{\delta }S^{\sigma +1} \end{array}$$ (20)

The problem (20) is a signomial GP problem with 5 degrees of difficulty.

We have *n* = 11 & *m* = 5. So there *d* = 11-(5+1) = 5.

## Method of problem solving

We consider two parameters R_1_ and R_2_.

$$\text{Max}\;\Pi \left(P,Q,M,T,S\right)=R_{1}-R_{2}$$ (21)

$$\left\{kP^{1-\alpha }M^{\delta }S^{\sigma }+kr_{1}P^{-\alpha }M^{\delta }S^{\sigma }Q^{3}+kr_{3}P^{-\alpha }M^{\delta }S^{\sigma }Q+2^{-1}ir_{1}Q^{3}kP^{-\alpha }M^{\delta }S^{\sigma }T+2^{-1}ir_{3}QkP^{-\alpha }M^{\delta }S^{\sigma }T\right\}=R_{1}$$ (22)

$$\left\{kr_{2}P^{-\alpha }M^{\delta }S^{\sigma }Q^{2}+kP^{-\alpha }M^{\delta +1}S^{\sigma }+kaP^{-\alpha }M^{\delta }S^{\sigma }Q^{-1}+2^{-1}ir_{2}Q^{2}kP^{-\alpha }M^{\delta }S^{\sigma }T+2^{-1}kP^{1-\alpha }M^{\delta }S^{\sigma }\theta T+kP^{-\alpha }M^{\delta }S^{\sigma +1}\right\}=R_{2}$$ (23)

$$\begin{array}{l} \text{Max}\;\Pi \left(P,Q,M,T,S\right)\\ \;=\left\{kP^{1-\alpha }M^{\delta }S^{\sigma }+kr_{1}P^{-\alpha }M^{\delta }S^{\sigma }Q^{3}+kr_{3}P^{-\alpha }M^{\delta }S^{\sigma }Q+2^{-1}ir_{1}Q^{3}kP^{-\alpha }M^{\delta }S^{\sigma }T+2^{-1}ir_{3}QkP^{-\alpha }M^{\delta }S^{\sigma }T\right\}\\ \;-\left\{kr_{2}P^{-\alpha }M^{\delta }S^{\sigma }Q^{2}+kP^{-\alpha }M^{\delta +1}S^{\sigma }+kaP^{-\alpha }M^{\delta }S^{\sigma }Q^{-1}+2^{-1}ir_{2}Q^{2}kP^{-\alpha }M^{\delta }S^{\sigma }T+2^{-1}kP^{1-\alpha }M^{\delta }S^{\sigma }\theta T+kP^{-\alpha }M^{\delta }S^{\sigma +1}\right\} \end{array}$$ (24)

We propose a variable *Z* as follows:

MaxΠ(P,Q,M,T,S)=Z (25)

$$Z\leq R_{1}-R_{2}\to Z+R_{2}\leq R_{1}\to Z+R_{2}\leq Y\leq R_{1}$$ (26)

$$Z+R_{2}\leq Y\to ZY^{-1}+R_{2}Y^{-1}\leq 1$$ (27)

$$Y\leq R_{1}\to R_{1}Y^{-1}\geq 1$$ (28)

Also, we have:

$$\overset{N}{\underset{i=1}{\prod }}{\left(\frac{u_{i}}{a_{i}}\right)}^{a_{i}}\leq \overset{N}{\underset{i=1}{\sum }}u_{i}$$ (29)

$$\overset{N}{\underset{i=1}{\sum }}a_{i}=1$$ (30)

$${\left(\overset{N}{\underset{i=1}{\sum }}u_{i}\right)}^{-1}\leq \overset{N}{\underset{i=1}{\prod }}{\left(\frac{u_{i}}{a_{i}}\right)}^{-a_{i}}$$ (31)

For the first restriction, we have:

$$R_{1}=R_{11}+R_{12}+R_{13}+R_{14}+R_{15}$$ (32)

$$R_{1}Y^{-1}\geq 1\to R_{11}Y^{-1}+R_{12}Y^{-1}+R_{13}Y^{-1}+R_{14}Y^{-1}+R_{15}Y^{-1}\geq 1$$ (33)

$${\left(R_{11}Y^{-1}+R_{12}Y^{-1}+R_{13}Y^{-1}+R_{14}Y^{-1}+R_{15}Y^{-1}\right)}^{-1}\leq 1$$ (34)

$${\left(R_{11}Y^{-1}+R_{12}Y^{-1}+R_{13}Y^{-1}+R_{14}Y^{-1}+R_{15}Y^{-1}\right)}^{-1}\leq {\left(\frac{R_{11}Y^{-1}}{L_{1}}\right)}^{-L_{1}}{\left(\frac{R_{12}Y^{-1}}{L_{2}}\right)}^{-L_{2}}{\left(\frac{R_{13}Y^{-1}}{L_{3}}\right)}^{-L_{3}}{\left(\frac{R_{14}Y^{-1}}{L_{4}}\right)}^{-L_{4}}{\left(\frac{R_{15}Y^{-1}}{L_{5}}\right)}^{-L_{5}}$$ (35)

$$L_{i}=\frac{R_{1i}}{\sum_{i=1}^{5}R_{1i}}$$ (36)

We have:

$$R_{11}=kP^{1-\alpha }M^{\delta }S^{\sigma }$$ (37)

$$R_{12}=kr_{1}P^{-\alpha }M^{\delta }S^{\sigma }Q^{3}$$ (38)

$$R_{13}=kr_{3}P^{-\alpha }M^{\delta }S^{\sigma }Q$$ (39)

$$R_{14}=2^{-1}ir_{1}Q^{3}kP^{-\alpha }M^{\delta }S^{\sigma }T$$ (40)

$$R_{15}=2^{-1}ir_{3}QkP^{-\alpha }M^{\delta }S^{\sigma }T$$ (41)

And also:

$$W=\overset{5}{\underset{i=1}{\sum }}R_{1i}$$ (42)

$$L_{1}=\frac{\left(kP^{1-\alpha }M^{\delta }S^{\sigma }\right)}{W}$$ (43)

$$L_{2}=\frac{\left(kr_{1}P^{-\alpha }M^{\delta }S^{\sigma }Q^{3}\right)}{W}$$ (44)

$$L_{3}=\frac{\left(kr_{3}P^{-\alpha }M^{\delta }S^{\sigma }Q\right)}{W}$$ (45)

$$L_{4}=\frac{\left(2^{-1}ir_{1}Q^{3}kP^{-\alpha }M^{\delta }S^{\sigma }T\right)}{W}$$ (46)

$$L_{5}=\frac{\left(2^{-1}ir_{3}QkP^{-\alpha }M^{\delta }S^{\sigma }T\right)}{W}$$ (47)

So we have:

$${\left(\frac{R_{11}Y^{-1}}{L_{1}}\right)}^{-L_{1}}{\left(\frac{R_{12}Y^{-1}}{L_{2}}\right)}^{-L_{2}}{\left(\frac{R_{13}Y^{-1}}{L_{3}}\right)}^{-L_{3}}{\left(\frac{R_{14}Y^{-1}}{L_{4}}\right)}^{-L_{4}}{\left(\frac{R_{15}Y^{-1}}{L_{5}}\right)}^{-L_{5}}\leq 1$$ (48)

For the second restriction, we have:

$$Z+R_{2}\leq Y\to ZY^{-1}+R_{2}Y^{-1}\leq 1$$ (49)

$$ZY^{-1}+R_{21}Y^{-1}+R_{22}Y^{-1}+R_{23}Y^{-1}+R_{24}Y^{-1}+R_{25}Y^{-1}+R_{26}Y^{-1}\leq 1$$ (50)

Finally, we have:

$$\begin{array}{l} \text{Max}\;Z\;\text{or}\;\text{Min}\;Z^{-1}\\ {\left(\frac{R_{11}Y^{-1}}{L_{1}}\right)}^{-L_{1}}{\left(\frac{R_{12}Y^{-1}}{L_{2}}\right)}^{-L_{2}}{\left(\frac{R_{13}Y^{-1}}{L_{3}}\right)}^{-L_{3}}{\left(\frac{R_{14}Y^{-1}}{L_{4}}\right)}^{-L_{4}}{\left(\frac{R_{15}Y^{-1}}{L_{5}}\right)}^{-L_{5}}\leq 1\\ ZY^{-1}+R_{21}Y^{-1}+R_{22}Y^{-1}+R_{23}Y^{-1}+R_{24}Y^{-1}+R_{25}Y^{-1}+R_{26}Y^{-1}\leq 1 \end{array}$$ (51)

Therefore, we obtain:

$$\begin{array}{l} \mathrm{Min}Z^{-1}\\ {\left(\frac{kP^{1-\alpha }M^{\delta }S^{\sigma }Y^{-1}}{L_{1}}\right)}^{-L_{1}}{\left(\frac{kr_{1}P^{-\alpha }M^{\delta }S^{\sigma }Q^{3}Y^{-1}}{L_{2}}\right)}^{-L_{2}}{\left(\frac{kr_{3}P^{-\alpha }M^{\delta }S^{\sigma }QY^{-1}}{L_{3}}\right)}^{-L_{3}}\\ {}*{\left(\frac{2^{-1}ir_{1}Q^{3}kP^{-\alpha }M^{\delta }S^{\sigma }TY^{-1}}{L_{4}}\right)}^{-L_{4}}{\left(\frac{2^{-1}ir_{3}QkP^{-\alpha }M^{\delta }S^{\sigma }TY^{-1}}{L_{5}}\right)}^{-L_{5}}\leq 1\\ ZY^{-1}+kr_{2}P^{-\alpha }M^{\delta }S^{\sigma }Q^{2}Y^{-1}+kP^{-\alpha }M^{\delta +1}S^{\sigma }Y^{-1}+kaP^{-\alpha }M^{\delta }S^{\sigma }Q^{-1}Y^{-1}+2^{-1}ir_{2}Q^{2}kP^{-\alpha }M^{\delta }S^{\sigma }TY^{-1}+2^{-1}kP^{1-\alpha }M^{\delta }S^{\sigma }\theta TY^{-1}+kP^{-\alpha }M^{\delta }S^{\sigma +1}Y^{-1}\leq 1 \end{array}$$ (52)

The final model obtains as follows:

$$\begin{array}{l} \text{Min}\;Z^{-1}\\ {\left(kP^{1-\alpha }M^{\delta }S^{\sigma }Y^{-1}\right)}^{-L_{1}}{\left(kr_{1}P^{-\alpha }M^{\delta }S^{\sigma }Q^{3}Y^{-1}\right)}^{-L_{2}}{\left(kr_{3}P^{-\alpha }M^{\delta }S^{\sigma }QY^{-1}\right)}^{-L_{3}}\\ {}*{\left(2^{-1}ir_{1}Q^{3}kP^{-\alpha }M^{\delta }S^{\sigma }TY^{-1}\right)}^{-L_{4}}{\left(2^{-1}ir_{3}QkP^{-\alpha }M^{\delta }S^{\sigma }TY^{-1}\right)}^{-L_{5}}L_{1}{}^{L_{1}}L_{2}{}^{L_{2}}L_{3}{}^{L_{3}}L_{4}{}^{L_{4}}L_{5}{}^{L_{5}}\leq 1\\ ZY^{-1}+kr_{2}P^{-\alpha }M^{\delta }S^{\sigma }Q^{2}Y^{-1}+kP^{-\alpha }M^{\delta +1}S^{\sigma }Y^{-1}+kaP^{-\alpha }M^{\delta }S^{\sigma }Q^{-1}Y^{-1}\\ +2^{-1}ir_{2}Q^{2}kP^{-\alpha }M^{\delta }S^{\sigma }TY^{-1}+2^{-1}kP^{1-\alpha }M^{\delta }S^{\sigma }\theta TY^{-1}+kP^{-\alpha }M^{\delta }S^{\sigma +1}Y^{-1}\leq 1\\ P>0,C>0,M>0,Q>0,S>0,i>0,a>0,k>0,r_{i}>0,T>0,\theta >0,Z>0 \end{array}$$ (53)

## Solution approach, numerical experiment and sensitivity analyses

### Solution approach

Therefore, the model (53) is a posynomial GP with five degrees of difficulty (Duffin et al. [5]) and to find the optimal solution of the problem, the CVX modeling system (Grant & Boyd [39]) is used.

### A numerical example

Consider a particular manufacturer who plans to determine the production lot-size, total revenue, production cost, marketing cost, set-up cost, inventory holding cost, inventory deteriorating cost and after-sales service cost. For this example, assume that the parameters are as follows:

*k* = 1000000; *α* = 1.25; *δ* = 0.003; *σ* = 0.002; *a* = 3; Moreover, assume production cost is calculated as follows. *r*_*1*_ = 1.0058; *r*_*2*_ = 8.2637; *r*_*3*_ = 8.3788; *i* = 1; *θ* = 0.001 the implementation of CVX on the proposed GP model of this paper yields Π* = 202501.825; Q* = 0.7245; P* = 35.8572; M* = 0.0541; S* = 0.0361; T* = 0.0032.

All computations are coded in MATLAB software, and they are available in supplementary file as S1 M-File.

One of the primary assumptions in most optimal pricing methods is that the production cost is a non-increasing function of lot-size. This assumption does not hold for many real-world applications since the cost of unit production may have decreased trend up to a certain level and then it starts increasing for many reasons such as an increase in wages, depreciation, etc. However, the production cost eventually has a declining trend that can be demonstrated in terms of cubic function and the resulted optimal pricing model can be modeled in Geometric Programming (GP).

**Fig 1:**
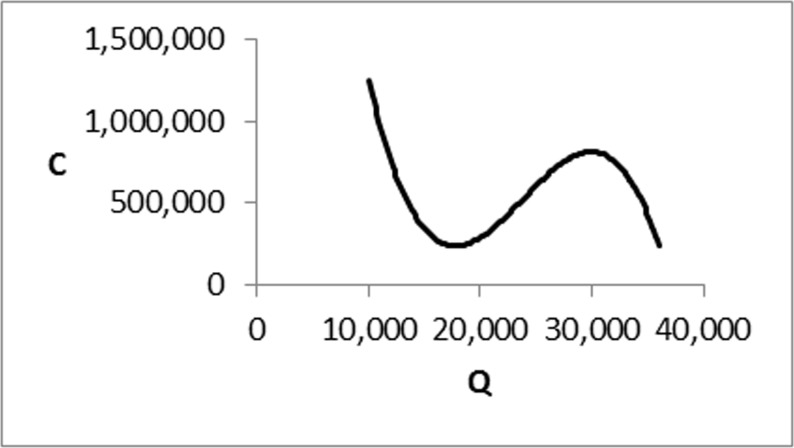
The cost of production (*C*) versus lot-size (*Q*).

It can be observed from Fig 1 that as the lot-size (*Q*) value increases, production cost decreases except for a specified period (Range 18,000 to 30,000). The reason for this issue goes to the difference between the cost of production in the day and night. It should be noted that, some expenditures during the night time such as wage rates, costs of lighting and heating workspaces, transportation costs and etc., are higher comparing to daytime.

### Sensitivity analyses

In order to understand the behavior of the proposed method when different parameters change, we have solved the proposed model different times by changing the parameters as Table 1 shows the computational results.

**Table 1:** Sensitivity analysis on parameters.

Parameter		P*	Q*	M*	S*	T*	Π*
*α*	1.25	35.857196	0.724421	0.05405	0.03607	0.0032	202501.823
	1.5	24.879012	0.70785	0.028735	0.019175	0.001396	75834.9123
	1.75	20.750169	0.700358	0.019703	0.013147	0.000936	31908.8298
	2	18.542841	0.696025	0.01502	0.010022	0.00074	14247.8887
	2.25	17.159121	0.693192	0.012147	0.008105	0.000635	6601.51812
*δ*	0.003	35.857196	0.724421	0.05405	0.03607	0.0032	202501.8253
	0.006	36.163411	0.72484	0.109369	0.036465	0.003211	201551.45
	0.009	36.479201	0.725253	0.16582	0.036854	0.003274	200931.5019
	0.012	36.801726	0.725672	0.2235	0.037254	0.003341	200529.6091
	0.015	37.131209	0.726096	0.282448	0.037662	0.003409	200292.5661
*σ*	0.002	35.857196	0.724421	0.05405	0.03607	0.0032	202501.8253
	0.004	36.059879	0.724702	0.054499	0.072658	0.003202	201701.749
	0.006	36.26801	0.724976	0.054885	0.109758	0.003237	201121.3871
	0.008	36.479267	0.725252	0.055277	0.147395	0.003281	200683.5531
	0.01	36.693355	0.725532	0.055675	0.185576	0.003319	200353.8752
*k*	1,000,000	35.857196	0.7244221	0.05405	0.03607	0.0032	202501.8253
	2,000,000	35.856886	0.724423	0.054049	0.03607	0.003236	405005.9915
	3,000,000	35.855835	0.724425	0.054046	0.036069	0.00332	607508.6013
	4,000,000	35.854769	0.724427	0.054042	0.036069	0.00347	810011.2513
	5,000,000	35.852764	0.724431	0.054038	0.036021	0.003559	1014334.218
*i*	1	35.857196	0.724421	0.05405	0.03607	0.0032	202501.8253
	2	35.985128	0.723519	0.054228	0.036155	0.0031457	201960.3424
	3	36.042318	0.723114	0.054267	0.036182	0.003016	201729.3087
	4	36.072942	0.722881	0.054297	0.036201	0.002912	201601.7504
	5	36.093898	0.722732	0.054309	0.036209	0.002653	201518.6155
*a*	3	35.857196	0.724421	0.05405	0.03607	0.0032	202501.8253
	4	44.420553	0.789264	0.068766	0.045889	0.005587	197388.6881
	5	55.239215	0.855127	0.070184	0.056837	0.007672	192934.3563
	6	66.050972	0.910892	0.081596	0.067779	0.009765	187491.3371
	7	78.7256821	0.980145	0.092869	0.0785291	0.011728	182127.2674
*θ*	0.001	35.857196	0.724421	0.05405	0.03607	0.0032	202501.8253
	0.002	35.8795	0.724276	0.054067	0.036082	0.002713	202406.6889
	0.003	35.90193	0.724106	0.054099	0.036105	0.002286	202304.7828
	0.004	35.926891	0.723929	0.054163	0.036121	0.001944	202199.0369
	0.005	35.955387	0.723729	0.054206	0.036141	0.001724	202081.2688

In order to have a better understanding of the behavior of the algorithm used for the model presented in this paper, we need to carefully analyze the behavior of the optimal solution with regard to changes in each parameter of the GP model. In the following sub-sections, we separately evaluate and monitor the effects of changes in various parameters of the proposed power functions on the optimal values of decision variables. However, due to high degrees of nonlinearities of the model and interdependencies of some decision variables, it is not always possible to expect a certain behavior of the decision variables.

### Changes in parameters of the demand function

**Fig 2:**
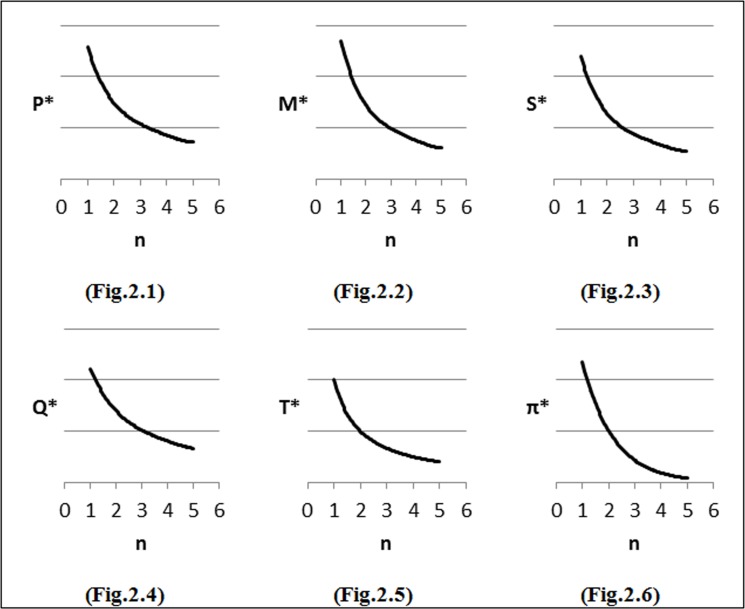
Effects of changes in (*α*) on decision variables.

To perform the first step of sensitivity analysis, we considered changes in the input parameters of the demand function on the optimal solution and the results are shown in Fig 2–Fig 7. In all figures, the horizontal axis represents different numbers of instances (n) and the vertical axis shows the optimal selling price per unit (P*), optimal marketing expenditure per unit (M*), optimal production lot-size (Q*), optimal after-sales service expenditure per unit (S*), optimal duration of each period (T*) and optimal profit (Π*).

**Fig 3:**
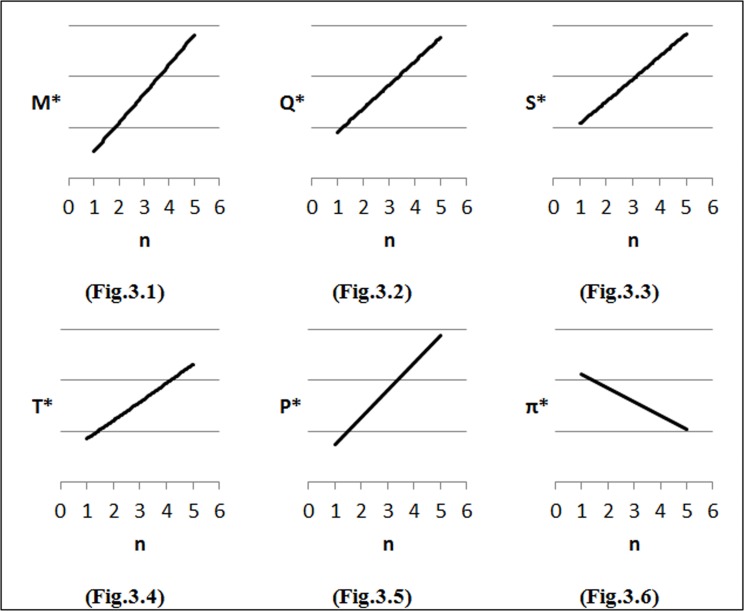
Effects of changes in (*δ*) on decision variables.

**Fig 4:**
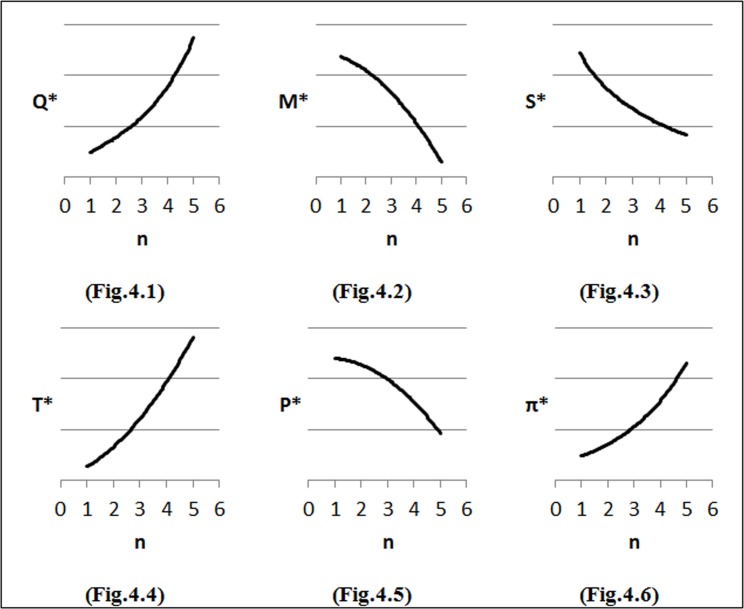
Effects of changes in (*k*) on decision variables.

**Fig 5:**
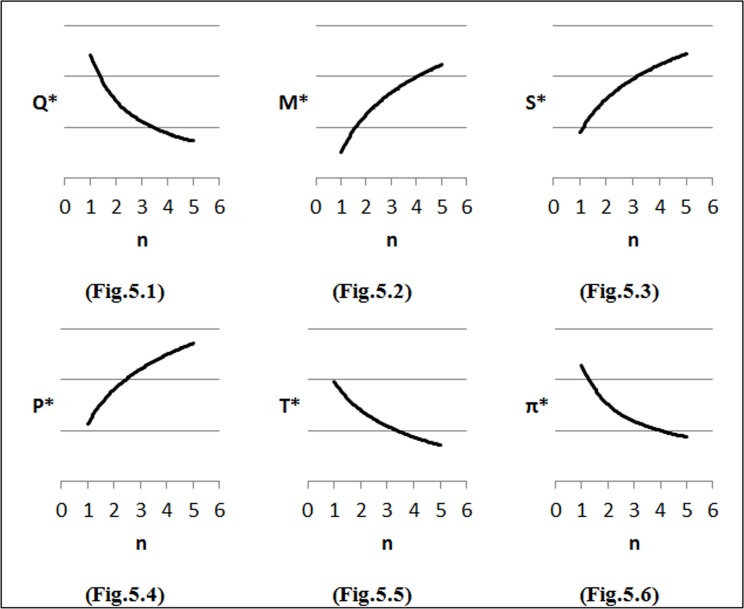
Effects of changes in (*i*) on decision variables.

**Fig 6:**
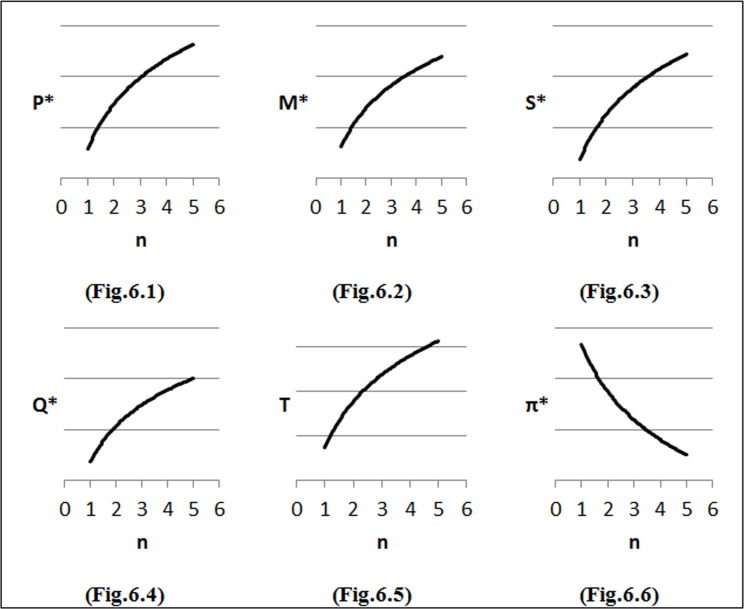
Effects of changes in (*a*) on decision variables.

**Fig 7:**
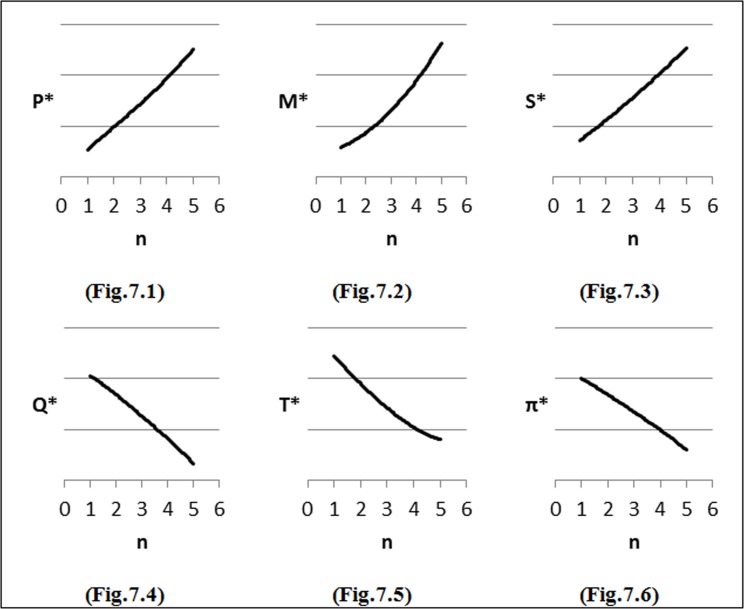
Effects of changes in (*θ*) on decision variables.

#### Effects of changes in *α* on optimal solution

Firstly, the impacts of changes in values of *α* on the optimal solution are considered. We investigate different values of *α* as follows: *α* = *α*_*0*_ + 0.25, where *α*_*0*_ = 1. We observe from Fig 2.1 that as *α* increases, the optimal selling price (*P*) decreases. This is because of an increase in price elasticity to demand that made the company to decrease its product price in order to maintain the desired market share. As shown in Fig 2.2 and Fig 2.3, with the increase in *α*, the optimal marketing expenditures and after-sales service cost decrease. Fig 2.4 and Fig 2.5 illustratethe behavior of the lot-size (*Q*) with the consideration of changes in the demand elasticity of price (*α*) and it can be seen that when the value of *α* increases, the value of lot-size (*Q*) decreases and will reduce *T*. As shown in Fig 2.6, generally when customers have highly price sensitivity, the company faces a high risk of losing some of them and this can lead to declining profit. Though through a reduction in the product’s price, the market share can be maintained while, the sales revenue will fall. Therefore the company can reduce its total production and inventory holding costs and hence faces huge losses in profit by reducing the lot-size (*Q*).

#### Effects of changes in *δ* on optimal solution

In this section, we examine the effects of different values of *δ* i.e. *δ* = *δ*_0_ + 0.003, where *δ*_0_ = 0 on profit maximization. As can be seen from Fig 3.1, by increasing marketing expenditure elasticity to demand (*δ*), the marketing expenditure (*M*) increases. Fig 3.2 illustrates the behavior of the lot-size (*Q*) with consideration of changes in the demand elasticity of marketing (*δ*). From this figure, it can be seen that when the value of *δ* increases, the value of lot-size (*Q*) increases. As shown in Fig 3.3 and 3.4, an increase in demand and lot-size (*Q*) result in an increase in after-sales service expenditure (*S*) and *T*. In order to offset the increase in costs, the selling price per unit (*P*) increases (Fig 3.5). As to be concluded, by increasing expenditure, total profit decreases, as well (Fig 3.6).

#### Effects of changes in *σ* on optimal solution

We have also investigated different values of *σ* on profitability, when *σ* = *σ*_*0*_ + 0.002, with *σ*_0_ = 0. The behavior of after-sales service expenditure elasticity to demand (σ) is similar to marketing expenditure elasticity to demand (*δ*). After-sales service expenditure can be included many items such as on-time delivery, customer support, product satisfaction, and repurchase intent.

#### Effects of changes in *k* on optimal solution

We have considered the effects of different values of *k* on profitability based on *k* = *k*_0_ + 1000000, where *k*_0_ = 0. As seen From Fig 4.1, the demand maintains upward trend with an increase in *k*. The increase in demand results in an increase in production lot-size (*Q*). From Fig 4.2–4.4 as *k* increases, duration of each period (*T*) increases and the marketing expenditure (*M*) and after-seals service cost (*S*) decrease. According to Fig 4.5 and 4.6, the company has to decrease its selling price per unit (*P*) in order to maintain the desired market share and increase its profit.

### Effects of changes in *i* on optimal solution

We have also examined diverse estimations of *i* as follows: *i* = *i*_*0*_ + 0.25, where *i*_*0*_ = 0. We can observe from Fig 5.1, when inventory holding cost increases, Lot-size (*Q*) should be decreased with the purpose of diminish inventory. Also, increasing in marketing (*M*) and after-sales service expenditure (*S*) will lead to increase product sale (Fig 5.2 and 5.3). In this situation, the company has to increase selling price in order to offset costs and increase the revenue (Fig 5.4). From Fig 5.5 it can be seen that by decreasing Lot-size and increasing demand, duration of each period (*T*) will be decreased. Finally, as expenditures increase the profit decrease (Fig 5.6).

### Effects of changes in *a* on optimal solution

We consider different values of *a* based on *a* = *a*_*0*_+1, where *a*_*0*_ = 2. According to Fig 6.1, when set-up costs of the production (*a*) increases, the company has to increase selling price (*P*) in order to offset costs and increase revenue. As shown in Fig 6.2 and 6.3, by increasing marketing expenditure (*M*) and after-sales service cost (*S*) (more advertising and better quality service) the amount of sell then increase. In this situation by increasing demand, Lot-size (*Q*) and duration of each period (*T*) increase (Fig 6.4 and 6.5). That is clear as expenditure increase the profit decrease (Fig 6.6).

### Effects of changes in *θ* on optimal solution

We have also considered *θ = θ*_*0*_ + 0.001, where *θ*_*0*_ = 0, for different values of *θ* to analyze the profit function. As shown in Fig 7.1, the price of the product (*P*) should be increased with the purpose of offset company losses when inventory deteriorating rates (*θ*) increase. As can be seen from Fig 7.2 and 7.3, the costs of advertising (*M*) and after-sales service (*S*) increase in order to increase the amount of sale, advertising and after-sales service. Fig 7.4 and 7.5 illustrate a decrease in the lot-size (*Q*) and duration of each period (*T*) when inventory deteriorating rates increase for avoiding increase in inventory deteriorating costs. Last of all, an increase in costs typically causes a decrease in profits (Fig 7.6).

## Conclusions

This paper has concentrated on a single-product inventory system for deterioration items. We have presented an algorithm to determine the optimal price, lot-size production, marketing expenditure, after-sales service expenditure and duration of each period. This model contains total revenue, production cost, marketing cost, set-up cost, inventory holding cost, inventory deteriorating cost and after-sales service cost.

We have considered production rate to be a linear function of demand. The model presented in this paper also considered demand to be a function of price, marketing and after-sales service. The resulting problem formulation is a signomial problem with five degrees of difficulty. We have used Geometric Programming to determine the optimal solution of the proposed model. Therefore, the CVX optimization toolbox, run in the MATLAB environment, is applied in order to solve this non-linear optimization problem. Numerical examples have been used to present the implementations of our algorithm. One of the extensions of our model is to consider the proposed model for a multi-product case with some linear constraints. Such a problem cannot be easily converted in to a transformed posynomial problem and would be an interesting area for future research.

## Supporting information

S1 M-File The MATLAB code used in the paper for numerical operations is uploaded in the site: (https://sites.google.com/site/gpnonlinearoptimization/). (M)
